# A multicenter investigation of 2,773 cases of bloodstream infections based on China antimicrobial surveillance network (CHINET)

**DOI:** 10.3389/fcimb.2022.1075185

**Published:** 2022-12-15

**Authors:** Fupin Hu, Lili Yuan, Yang Yang, Yuanhong Xu, Ying Huang, Yunjian Hu, Xiaoman Ai, Chao Zhuo, Danhong Su, Bin Shan, Yan Du, Yunsong Yu, Jie Lin, Ziyong Sun, Zhongju Chen, Yingchun Xu, Xiaojiang Zhang, Chuanqing Wang, Leiyan He, Yuxing Ni, Yibo Zhang, Dongfang Lin, Demei Zhu, Yingyuan Zhang

**Affiliations:** ^1^Institute of Antibiotics, Huashan Hospital, Fudan University, Shanghai, China; ^2^Key Laboratory of Clinical Pharmacology of Antibiotics, National Health Commission, Shanghai, China; ^3^Department of Hospital Infection Management, Huashan Hospital, Fudan University, Shanghai, China; ^4^Clinical Microbiology Laboratory, The First Affiliated Hospital of Anhui Medical University, Hefei, China; ^5^Clinical Microbiology Laboratory, Beijing Hospital, Beijing, China; ^6^Department of Infectious Disease, The First Affiliated Hospital of Guangzhou Medical University, Guangzhou, China; ^7^Clinical Microbiology Laboratory, The First Affiliated Hospital of Guangzhou Medical University, Guangzhou, China; ^8^Clinical Microbiology Laboratory, The First Affiliated Hospital of Kunming Medical University, Kunming, China; ^9^Department of Infectious Disease, Sir Run Run Shaw Hospital, Affiliated to Zhejiang University School of Medicine, Hangzhou, China; ^10^Clinical Microbiology Laboratory, Sir Run Run Shaw Hospital, Affiliated to Zhejiang University School of Medicine, Hangzhou, China; ^11^Clinical Microbiology Laboratory, Tongji Hospital Affiliated to Tongji Medical College of Huazhong University of Science and Technology, Wuhan, China; ^12^Clinical Microbiology Laboratory, Peking Union Medical College Hospital, Beijing, China; ^13^Clinical Microbiology Laboratory, Children’s Hospital of Fudan University, Shanghai, China; ^14^Clinical Microbiology Laboratory, Ruijin Hospital Affiliated to Shanghai Jiaotong University School of Medicine, Shanghai, China; ^15^Department of Hospital Infection Management, Ruijin Hospital Affiliated to Shanghai Jiaotong University School of Medicine, Shanghai, China

**Keywords:** bloodstream infection, community-acquired, hospital-acquired, multicenter investigation, antimicrobial susceptibility testing

## Abstract

**Background:**

Bloodstream infections (BSIs), especially hospital-acquired BSIs, are a major cause of morbidity and mortality. However, the details about the pathogens and antimicrobial resistance profile of BSIs across China are still lacking.

**Methods:**

An investigation was conducted in 10 large teaching hospitals from seven geographic regions across China in 2016 based on China Antimicrobial Surveillance Network (CHINET) to profile the clinical and etiological features of BSIs.

**Results:**

A total of 2,773 cases of BSIs were identified, a majority (97.3%) of which were monomicrobial. Overall, 38.4% (1,065/2,773) were community-acquired BSIs (CABSIs), and 61.6% (1,708/2,773) were hospital-acquired BSIs (HABSIs). Of the 2,861 pathogenic BSI isolates, 67.5% were Gram-negative bacteria, 29.6% were Gram-positive bacteria, and 2.9% were fungi. The top BSI pathogens were *Escherichia coli*, *Klebsiella pneumoniae*, coagulase-negative *Staphylococci* (CNS), *Staphylococcus aureus*, *Enterococci*, and *Acinetobacter baumannii*. *Escherichia coli* and *K. pneumoniae* isolates showed low susceptibility to penicillins, cephalosporins (except ceftazidime and cefepime), and ampicillin-sulbactam (13.1%–43.4% susceptible); moderate susceptibility (about 60% susceptible) to ceftazidime, cefepime, and aztreonam; and high susceptibility (>90%) to β-lactam/β-lactamase inhibitor combinations other than ampicillin-sulbactam, except *K. pneumoniae* strains to piperacillin-tazobactam (59.2% susceptible). HABSIs were associated with significantly higher prevalence of carbapenem-resistant and extended-spectrum β-lactamases-producing *K. pneumoniae*, methicillin-resistant *S. aureus*, methicillin-resistant CNS, and ampicillin-resistant *Enterococci* than CABSIs. Overall, 42.0% of the BSI due to *S. aureus* strains were resistant to methicillin.

**Conclusions:**

The findings about BSIs in teaching hospitals across China add more scientific evidence to inform the appropriate management of the disease.

Bloodstream infection (BSI) is one of the well-recognized critical infectious diseases. The use of invasive procedures (e.g., intravenous catheters, mechanical ventilation, and dialysis), medications (e.g., immunosuppressants and biological products), and the growing population of immunocompromised or immunodeficient individuals have contributed to the high incidence of infections, including BSIs. The extensive and intensive use of broad-spectrum antibiotics has made the situation of antimicrobial resistance worse, and so the prevalence of drug-resistant bacteria is also increasing in BSIs (Yang et al., 2019). The antibiotic-resistant bacterial infections are difficult to manage, are associated with high mortality, and pose a great threat to health and life, which have caused heavy social and economic burden (Rodríguez-Créixems et al., 2008; Cassini et al., 2019). The widespread of carbapenem-resistant Gram-negative bacilli and other multidrug-resistant or extensively drug-resistant strains in recent years has brought great challenges to the antimicrobial treatment and narrowed the empirical treatment options for various infectious diseases, including BSIs (Hu et al., 2018).

Timely and accurate etiological diagnosis and early and appropriate antimicrobial treatment are crucial to optimal outcomes of BSIs (Rodríguez-Baño et al., 2010). The appropriateness of initial empirical treatment is dependent on the knowledge of local BSI pathogens and their resistance patterns. However, the data of large series of BSIs are still lacking in China even though such data are available internationally, which inform the changing BSI pathogens and their resistance profiles over time. The ongoing antimicrobial resistance surveillance in China only reported the BSI data in individual hospitals. Some additional reports on BSI cases are also available but of small sample size (Li et al., 2013; Chen et al., 2016; Zhou Y, et al., 2021; Zhou J, et al., 2021). Therefore, it is urgently needed to fully understand the distribution and resistance of BSI pathogens in China for better management of the disease. This investigation was based on the data from China Antimicrobial Surveillance Network (CHINET) (www.chinets.com) to conduct a prospective multicenter survey on BSIs for the first time in China. The findings will be conducive to appropriate empirical and targeted antimicrobial treatment of BSIs and inform the strategies for etiological diagnosis and antimicrobial therapy of BSIs in China.

## Methods

### Study design

Ten large teaching hospitals, including a children’s hospital, were selected from the members of CHINET program to participate in this investigation, covering seven provinces or municipalities across China. The participating hospitals ranged in size from 689 to 6,000 beds (2,576 on average). The patients with positive blood culture during treatment in any of the participating hospitals, outpatient clinic, or emergency department from 1 January to 31 December 2016 were enrolled in this study. BSI diagnosis is described according to the diagnostic criteria. Clinical data were retrieved from the hospital information system of each study center, including demographic data, clinical service at the onset of BSI, predisposing factors, disease status, anti-infective treatment, and outcomes. The pathogenic bacteria collected from each center were sent to the central laboratory, i.e., Clinical Microbiology Laboratory of Institute of Antibiotics, Huashan Hospital Affiliated to Fudan University, for species re-identification and re-testing of antimicrobial susceptibility. The distribution and resistance profile of pathogenic bacteria were analyzed.

The study protocol was reviewed and approved by the Institutional Review Board (IRB) of Huashan Hospital, Fudan University. The remaining nine participating centers (hospitals) agreed with the conclusion of the IRB of Huashan Hospital. Informed consent was waived for all the patients.

### Study population

The patients with confirmed BSI were considered as the study population. The eligible patients were enrolled at each center if they met the diagnostic criteria for BSI (Ministry of Health of People’s Republic of China, 2001; Horan et al., 2008). The patients were excluded if the positive blood culture was due to skin contaminant or colonizing bacteria or medical history data were incomplete or missing. The first isolates from individual patients were collected and analyzed. Duplicate organisms from one patient were not collected and analyzed. Different organisms from various periods were collected to evaluate as independent episode or co-infection.

### BSI diagnosis

BSI diagnosis was confirmed if the patient had one or more blood cultures positive for a pathogenic bacterium; and any of the symptoms or signs such as fever ≥38°C or <36°C, chills, or hypotension (systolic blood pressure ≤90 mmHg); and any of the following laboratory abnormalities: peripheral blood leukocytosis, or neutrophilia, or neutrophil left shift, or increased C-reactive protein (CRP) or procalcitonin (PCT). For the skin contaminant or colonizing bacteria (e.g., coagulase-negative *Staphylococci*), BSI was not considered unless the same bacterial strain was cultured from the blood samples taken from two different sites, or the bacterial strain isolated from blood was the same as that isolated from other body sites or intravascular catheter specimen. The BSI episodes were excluded if they were considered relapse rather than a separate infection.

BSIs were further differentiated as hospital acquired (HABSI) or community acquired (CABSI) according to where the BSI was acquired (Horan et al., 2008). HABSI was considered in a patient who had a positive blood culture and acquired BSI at least 48 h after admission or <48 h after discharge and he/she had no infection in the incubation period of infection at time of hospital admission. CABSI was considered in a patient who had a positive blood culture and acquired BSI in community or within 48 h after admission.

### Microbiological methods

Blood cultures were sampled, according to predefined indications, in BacT/ALERT FA Plus and PF Plus (bioMérieux, Marcy-l’Etoile, France) blood culture bottles. Isolates retrieved from the blood culture bottles were processed locally and later shipped to Huashan Hospital for reference identification [matrix-assisted laser desorption/ionization time-of-flight spectrometry (MALDI-TOF), bioMérieux)] and antimicrobial susceptibility testing (broth microdilution method). All microbiological methods were consistent with current Clinical and Laboratory Standards Institute (CLSI) recommendations. The isolates were stored at −70°C for further tests. The results were interpreted according to the breakpoints recommended by CLSI in 2018 (CLSI, 2018b; CLSI, 2018b).

### Statistical analysis

The antimicrobial susceptibility data were processed and analyzed using WHONET 5.6 software. The measurement data of clinical variables were compared statistically by two-sided *t*-test *via* SPSS 22.0 software. The enumeration data were compared by chi-square test. *p*<0.05 was considered statistically significant in univariate analysis.

## Results

### Patient and disease characteristics

A total of 6,235 blood-culture isolates were isolated from 5,613 patients from 10 participating cites during CHINET 2016. A total of 1,966 episodes were excluded as contaminants, and the majority were isolated with single or dual coagulase-negative *Staphylococci*. There were 874 episodes that were excluded as inadequate information. A total of 2,773 patients were identified with BSI diagnosis during the study period. Overall, 2,861 pathogenic strains were isolated, including monomicrobial BSI in 2,697 (97.3%) cases and polymicrobial BSI in 76 (2.7%) cases. Overall, 61.6% (1,709/2,773) of the BSIs were hospital acquired, and 38.4% (1,064/2,773) were community acquired. About 8.9% (248/2,773) of the BSIs occurred in outpatients or in patients visiting the emergency department and 91.1% (2,525/2,773) in hospitalized patients, of which 19.3% (488/2,525) stayed in the intensive care unit (ICU) and 80.7% (2,037/2,525) were treated in non-intensive care wards.

Most of the patients (86.2%, 2,390/2,773) were at least 18 years old. The primary site of infection was identified in 34.4% (955/2,773) of the cases. It was similar between HABSIs and CABSIs; besides, more CABSI patients also had urinary tract infection and biliary tract infection (**Table 1**).

**Table 1:** Demographic and clinical data compared between hospital- and community-acquired bloodstream infections.

Characteristic	Total (*n* = 2,773)	Hospital-acquired BSI (*n* = 1,709)	Community-acquired BSI (*n* = 1,064)	*p-*value
**Age**				
Newborns (≤ 28 days)	185 (6.7)	102 (6.0)	83 (7.8)	0.072
29 days to < 18 year	198 (7.1)	132 (7.7)	66 (6.2)	0.082
≥18 years to < 65 years	1,501 (54.1)	957 (56)	544 (51.1)	0.014
≥ 65 years	889 (32.1)	518 (30.3)	371 (34.9)	0.006
**Sex**				
Male	1,614 (58.2)	1,021 (59.7)	593 (55.7)	0.040
Female	1,159 (41.8)	688 (40.3)	471 (44.3)	0.040
**Underlying disease**				
Solid tumor	395 (14.2)	285 (16.7)	110 (10.3)	<0.001
Hematological malignancies	209 (7.5)	168 (9.8)	41 (3.9)	<0.001
Diabetes mellitus	204 (7.4)	129 (7.5)	75 (7.0)	0.654
Coronary heart disease	66 (2.4)	49 (2.9)	17 (1.6)	0.040
Hypertension	227 (8.2)	183 (10.7)	44 (4.1)	<0.001
Cerebrovascular accident	78 (2.8)	59 (3.5)	19 (1.8)	0.006
Urinary tract infection and stones	155 (5.6)	95 (5.6)	60 (5.6)	0.932
Pulmonary infection	176 (6.3)	95 (5.6)	81 (7.6)	0.037
COPD	17 (0.6)	12 (0.7)	5 (0.5)	0.618
Cirrhosis	64 (2.3)	41 (2.4)	23 (2.2)	0.795
Premature infant	69 (2.5)	48 (2.8)	21 (2)	0.210
Respiratory distress	49 (1.8)	34 (2)	15 (1.4)	0.301
Hyperbilirubinemia	19 (0.7)	6 (0.4)	13 (1.2)	0.009
**Primary site of infection**				
Pulmonary infection	284 (10.2)	174 (10.2)	110 (10.3)	0.898
Urinary tract infection	202 (7.3)	102 (6)	100 (9.3)	0.001
Biliary tract infection	120 (4.3)	59 (3.5)	61 (5.7)	0.005
Intra-abdominal infection	79 (2.8)	57 (3.3)	22 (2.1)	0.060
Skin and soft tissue infection	32 (1.2)	17 (1)	15 (1.4)	0.362
Surgical incision infection	21 (0.8)	18 (1.1)	3 (0.3)	0.024
Liver abscess	21 (0.8)	7 (0.4)	14 (1.3)	0.011
Not identified	1,818 (65.6)	1,151 (67.3)	667 (62.7)	0.012

BSI, bloodstream infection; COPD, chronic obstructive pulmonary disease.

Data are number (%) unless otherwise specified.

Fever was the prominent symptom of BSIs, reported in 88.0% (1,504/1,709) of the HABSI cases and 84.2% (896/1,064) of the CABSI cases. Intravascular catheters and other invasive procedures were the most prevalent predisposing factor for HABSIs, significantly higher than the prevalence in CABSIs (**Table 2**).

**Table 2:** Clinical features and predisposing factors compared between hospital- and community-acquired bloodstream infections.

Characteristic	Hospital-acquired BSI (*n* = 1,709)	Community-acquired BSI (*n* = 1,064)	*p-*value
**Clinical feature**			
Fever	1,504 (88)	896 (84.2)	0.005
Hypotension	103 (6)	96 (9)	0.004
Shock	68 (4)	51 (4.8)	0.335
Multiple organ failure	55 (3.2)	26 (2.4)	0.249
**Predisposing factor**			
Intravascular catheter	885 (51.8)	237 (22.3)	<0.001
Other invasive procedures^a^	971 (56.8)	287 (27.0)	<0.001
Neutropenia, chemotherapy	352 (20.6)	198 (18.6)	0.203

BSI, bloodstream infection.

Data are number (%) unless otherwise specified.

a Including endotracheal intubation, ventilator, and various invasive puncture and drainage.

### BSI pathogens

Of the 2,861 strains, the top 10 pathogens were *E. coli*, *K. pneumoniae*, coagulase-negative *Staphylococci*, *S. aureus*, *Enterococci*, *A. baumannii*, *P. aeruginosa*, *Streptococcus*, *Enterobacter*, and *Candida* species (**Table 3**). The prevalence of *E. coli* and *S. aureus* was significantly higher in CABSIs than in HABSIs, while HABSIs were associated with a significantly higher proportion of *K. pneumoniae* and *A. baumannii* than CABSIs. A significantly higher proportion of *A. baumannii*, coagulase-negative *Staphylococci*, and *Enterococci* were identified in the BSI pathogens isolated from ICU patients compared with that from non-ICU patients, while *E. coli* was more frequently isolated from non-ICU patients (**Table 4**). *Enterococci* and coagulase-negative *Staphylococci* were significantly associated with old patients (≥65 years) than younger adults (18–65 years) (**Table 5**).

**Table 3:** Distribution of 2,861 strains of pathogens isolated from bloodstream infections.

Microorganism	Number	%	Microorganism	Number	%
**Gram-negative bacteria**	**1,931**	**67.5**	**Gram-positive bacteria**	**847**	**29.6**
***Enterobacterales***	**1,465**	**51.2**	***Staphylococcus***	**470**	**16.4**
*E. coli*	770	26.9	*S. aureus*	208	7.3
*K. pneumoniae*	464	16.2	*S. epidermidis*	84	2.9
Other *Klebsiella*	45	1.6	*S. haemolyticus*	64	2.2
*Enterobacter*	88	3.1	*S. hominis*	52	1.8
*Serratia*	34	1.2	*S. capitis*	25	0.9
*Salmonella*	29	1.0	Other CNS	32	1.1
*Citrobacter*	13	0.5	Other *Staphylococcus*	5	0.2
*Proteus*	14	0.5	*Enterococci*	**233**	**8.1**
Other *Enterobacterales*	8	0.3	*E. faecalis*	111	3.9
**Nonfermentative bacteria**	**430**	**15.0**	*E. faecium*	102	3.6
*A. baumannii*	162	5.7	Other *Enterococci*	20	0.7
Other *Acinetobacter*	8	0.3	***Streptococcus***	**128**	**4.5**
*P. aeruginosa*	133	4.6	*S. viridans*	72	2.5
*S. maltophilia*	53	1.9	*S. pneumoniae*	25	0.9
*Burkholderia*	25	0.9	*S. agalactiae*	20	0.7
*Achromobacter*	22	0.8	*S. pyogenes*	4	0.1
Other *Pseudomonas*	11	0.4	*S. lactis*	3	0.1
Other non-fermentative bacteria	16	0.6	*S. bovis*	2	0.1
***Aeromonas***	**15**	**0.5**	Other *Streptococcus*	2	0.1
***Haemophilus***	**4**	**0.1**	***Listeria***	**5**	**0.2**
**Other Gram-negative bacteria**	**17**	**0.6**	**Other Gram-positive bacteria**	**11**	**0.4**
**Fungus**	**83**	**2.9**			
*C. albicans*	20	0.7			
*C. parapsilosis*	17	0.6			
*C. glabrata*	17	0.6			
*C. tropicalis*	13	0.5			
Other *Candida*	14	0.5			
*Cryptococcus*	2	0.1			

CS, coagulase-negative Staphylococci. The bold values are organism groups and accumulated numbers of related species.

**Table 4:** Distribution of pathogens isolated from hospital-acquired versus community-acquired bloodstream infections and ICU versus non-ICU patients.

Microorganism	HABSI (*n* = 1,777)	CABSI (*n* = 1,084)	*p*-value	Microorganism	ICU (*n* = 518)	Non-ICU (*n* = 2,343)	*p-*value
*E. coli*	419 (23.6)	351 (32.4)	<0.001	*E. coli*	65 (12.5)	705 (30.1)	<0.001
*K. pneumoniae*	316 (17.8)	148 (13.7)	0.004	*K. pneumoniae*	91 (17.6)	373 (15.9)	0.357
*S. aureus*	115 (6.5)	94 (8.7)	0.032	*S. aureus*	31 (6.0)	178 (7.6)	0.756
CNS	167 (9.4)	92 (8.5)	0.421	CNS	63 (12.2)	196 (8.4)	<0.001
*Enterococci*	155 (8.7)	78 (7.2)	0.159	*Enterococci*	55 (10.6)	178 (7.6)	0.026
*A. baumannii*	125 (7.0)	37 (3.4)	<0.001	*P. aeruginosa*	29 (5.6)	104 (4.4)	0.250
*P. aeruginosa*	91 (5.1))	42 (3.9)	0.143	*A. baumannii*	61 (11.8)	101 (4.3)	<0.001
*E. cloacae*	61 (3.4)	25 (2.3)	0.091	*E. cloacae*	9 (1.7)	77 (3.3)	0.065

CABSI, community-acquired bloodstream infection; CNS, coagulase-negative Staphylococci; HABSI, hospital-acquired bloodstream infection.

Data are number (%) unless otherwise specified.

**Table 5:** Distribution of pathogens isolated from bloodstream infections in terms of age group.

Microorganism	≥65 years(*n*=912)	18 to <65 years(*n*=1,557)	*p-*value	Microorganism	29 days to 18 years(*n*=202)	Newborns (≤28 days)(*n*=190)	*p*-value
*E. coli*	281 (30.8)	424 (27.2)	0.059	*E. coli*	35 (17.3)	30 (15.8)	0.786
*K. pneumoniae*	136 (14.9)	272 (17.5)	0.104	*K. pneumoniae*	19 (9.4.)	38 (20)	0.004
*S. aureus*	57 (6.3)	127 (8.2)	0.095	*S. aureus*	14 (6.9)	11 (5.8)	0.684
CNS	97 (10.6)	123 (7.9)	0.023	*S. pneumoniae*	12 (5.9)	0 (0)	<0.001
*A. baumannii*	50 (5.5)	107 (6.9)	0.200	*S. maltophilia*	13 (6.4)	5 (2.6)	0.091
*Enterococci*	104 (11.4)	95 (6.1)	<0.001	*P. aeruginosa*	11 (5.4)	2 (1.1)	0.021
*P. aeruginosa*	48 (5.3)	71 (4.6)	0.437	CNS	10 (5.0)	6 (3.2)	0.449
*E. cloacae*	32 (3.5)	42 (2.7)	0.272	*S. agalactiae*	1 (0.5)	12 (6.3)	0.001

CNS, coagulase-negative Staphylococci.

### Antimicrobial susceptibility of BSI pathogens

About 60% of the *E. coli* and *K. pneumoniae* isolates were susceptible to ceftazidime, cefepime, and aztreonam, more than 90% susceptible to β-lactam/β-lactamase inhibitor combinations (excluding ampicillin-sulbactam), but only 13.1%–43.4% susceptible to penicillins, most cephalosporins, and ampicillin-sulbactam. However, *K. pneumoniae* strains showed a relatively lower resistance rate to piperacillin-tazobactam (59.2%) than penicillins, most cephalosporins, and ampicillin-sulbactam. About 41.3%–53.3% of the *E. coli* and *K. pneumoniae* isolates were susceptible to aminoglycosides (excluding amikacin), fluoroquinolones, and trimethoprim-sulfamethoxazole. Tigecycline and polymyxin E were highly active against *E. coli* and *K. pneumoniae* (>95% susceptible). More than half of the *E. coli* and *K. pneumoniae* isolates (60.7% and 57.9%) were resistant to cefotaxime. The *E. coli* isolates showed apparently lower resistance rate to imipenem than *K. pneumoniae* (0.25% *vs*. 33.2%). Approximately 8.6%–10.5% of the *P. aeruginosa* strains were resistant to piperacillin, piperacillin-tazobactam, carbapenems, and fluoroquinolones; 3.4%–8.6% resistant to ceftazidime, cefepime, ceftazidime-avibactam, aminoglycosides, and polymyxin E; and 15.5% resistant to aztreonam. More than 70% of the *A. baumannii* isolates were resistant to most of the antimicrobial agents tested, except polymyxin E (only 2.9% resistant), aminoglycosides, levofloxacin, ceftazidime, and trimethoprim-sulfamethoxazole (55.9%–66.2% resistant) (**Table 6**).

**Table 6:** Susceptibility of Gram-negative bacilli isolated from bloodstream infections to antimicrobial agents.

Antimicrobial agent	*E. coli* (*n*=746)			*K. pneumoniae* (*n*=442)			*P. aeruginosa* (*n*=120)			*A. baumannii* (*n*=148)		
	MIC_90_	R%	S%	MIC_90_	R%	S%	MIC_90_	R%	S%	MIC_90_	R%	S%
Ampicillin	>32	86.0	13.1	>32	NA	NA	NA	NA	NA	NA	NA	NA
Piperacillin	>128	79.5	15.6	>128	57.9	40.7	128	10.3	84.5	NA	NA	NA
Cefazolin	>32	62.7	37.3	>32	60.1	39.9	NA	NA	NA	NA	NA	NA
Cefuroxime	>64	60.1	38.4	>64	57.8	40.4	NA	NA	NA	NA	NA	NA
Cefotaxime	>32	60.7	39.0	>32	58.7	41.3	NA	NA	NA	>32	83.1	6.8
Ceftazidime	>32	27.8	67.3	>32	48.4	48.8	8	6.9	91.4	>32	66.2	32.4
Cefepime	>32	27.7	61.4	>32	38.9	56.7	8	5.2	91.4	>32	72.1	27.9
Aztreonam	128	37.6	52.9	>128	47.3	52.1	32	15.5	74.1	128	79.4	10.3
Imipenem	0.25	1.4	98	32	33.2	66.2	4	8.6	81.0	32	70.6	29.4
Meropenem	0.25	1.4	98.3	>16	34.1	65.3	8	10.5	84.2	>16	70.6	29.4
Ampicillin-sulbactam	>32	44.5	43.4	>32	52.6	43.4	NA	NA	NA	>32	74.3	22.9
Piperacillin-tazobactam	16/4	5.2	90.5	>128/4	39.0	59.2	32/4	8.6	87.9	>128	70.6	25.0
Cefoperazone-sulbactam	32/16	NA	NA	>128/64	NA	NA	64/32	NA	NA	128/64	NA	NA
Ceftazidime-avibactam	1/4	2.2	97.8	2/4	1.9	98.1	4/4	7	93	>32/4	NA	NA
Aztreonam-avibactam	0.5/4	NA	NA	2/4	NA	NA	NA	NA	NA	NA	NA	NA
Gentamicin	64	42.5	55.8	>128	40.2	58.4	4	8.6	91.4	>128	66.2	30.9
Amikacin	8	5.8	94.2	>128	21.4	78.1	8	6.9	93.1	>128	60.3	39.7
Ciprofloxacin	16	57.8	41.3	16	49.1	49.1	2	8.6	86.2	16	72.1	27.9
Levofloxacin	32	52.3	41.9	32	44.9	53.3	8	10.3	86.2	32	60.3	27.9
Trimethoprim-sulfamethoxazole	>32	56.9	43.1	>32	48.1	51.9	NA	NA	NA	>32	55.9	44.1
Tigecycline	0.5	0	98.6	1	0.9	95.8	NA	NA	NA	2	NA	NA
Polymyxin E	2	3.8	96.2	2	2.8	97.2	2	3.4	96.6	2	2.9	97.1

MIC, minimum inhibitory concentration (mg/L); NA, not available.

The prevalence of methicillin-resistant *S. aureus* (MRSA) was 42.0% in the *S. aureus* isolates. The prevalence of methicillin-resistant strains (MRCNS) was 93.7% in the CNS strains. None of the staphylococcal strains were resistant to vancomycin, but 1.5% of the MRCNS strains were resistant to linezolid (**Table 7**). Seven (7/102, 6.9%) of the *E. faecalis* strains were intermediate to linezolid but all susceptible to vancomycin. Three (3/111, 2.7%) of the *E. faecium* strains were resistant to vancomycin.

**Table 7:** Susceptibility of Gram-positive bacterial strains isolated from bloodstream infections to antimicrobial agents.

Antimicrobial agent	MRSA (*n*=79)			MSSA (*n*=108)			MRCNS (*n*=195)			MSCNS (*n*=38)			*Enterococci* (*n*=188)		
	MIC_90_	R%	S%	MIC_90_	R%	S%	MIC_90_	R%	S%	MIC_90_	R%	S%	MIC_90_	R%	S%
Penicillin	>16	100	0	8	91.9	8.1	>16	100	0	4	66.7	33.3	NA	NA	NA
Ampicillin	NA	NA	NA	NA	NA	NA	NA	NA	NA	NA	NA	NA	>128	44.1	55.9
Oxacillin	>8	100	0	1	0	100	>8	100	0	0.25	0	100	NA	NA	NA
Erythromycin	>64	72.4	20.7	>64	40.5	56.8	>64	82	17.5	>64	46.2	53.8	NA	NA	NA
Clindamycin	>64	44.8	55.2	64	16.2	81.1	>64	30	67.5	>64	12.8	87.2	NA	NA	NA
Tigecycline	0.5	0	100	0.25	0	100	0.5	0	100	0.5	0	100	0.5	NA	NA
Rifampicin	4	10.3	89.7	0.5	0	100	0.25	6	93	≤0.125	0	100	NA	NA	NA
Gentamicin	>64	55.2	41.4	32	16.2	83.8	32	33.5	55.5	8	7.7	87.2	NA	NA	NA
Levofloxacin	>32	69	31	8	10.8	86.5	>32	69	29	4	12.8	87.2	>64	53.2	46.8
Trimethoprim-sulfamethoxazole	≤0.25	0	100	0.5	0	100	4	18.5	81.5	2	2.6	97.4	NA	NA	NA
Linezolid	1	0	100	1	0	100	1	1.5	98.5	1	0	100	2	0	96.3
Vancomycin	1	0	100	1	0	100	1	0	100	1	0	100	2	1.6	98.4

MIC, minimum inhibitory concentration (mg/L); NA, not available.

### Prevalence of important resistant pathogens in different patient populations

Compared with CABSIs, HABSIs were associated with a higher proportion of resistant *K. pneumoniae* strains, especially carbapenem-resistant *K. pneumoniae* and ESBLs-producing *K. pneumoniae*. The prevalence of MRSA, MRCNS, and ampicillin-resistant *Enterococci* was significantly higher in HABSIs than in CABSIs. Compared with the strains isolated from non-ICU patients, the *K. pneumoniae*, *P. aeruginosa*, and *A. baumannii* isolated from ICU patients showed higher resistance rates, and the prevalence of carbapenem-resistant *E. coli* and MRSA was apparently higher. However, the prevalence of ESBL-producing *E. coli* was higher in the non-ICU patients compared with ICU patients. Most of the BSI isolates except *P. aeruginosa* and *A. baumannii* showed similar resistance level between old patients and young adults (**Table 8**).

**Table 8:** Prevalence of important resistant pathogens isolated from bloodstream infections in different patient populations.

Organisms	Category of BSI		*p*-value	ICU admission		*p*-value	Age group		*p*-value
	CABSI	HABSI		ICU	Non-ICU		Young adult (≥18 to <65)	Old patient(≥65)	
ESBLs+ eco	57.4 (183/319)	61.5 (224/364)	0.275	40.4 (23/57)	61.3 (384/626)	0.003	62.8 (245/390)	58.7 (138/235)	0.311
CR-eco	0.3 (1/319)	1.6 (6/364)	0.129	3.5 (2/57)	0.8 (5/626)	0.110	1.0 (4/390)	0.9 (2/235)	0.999
ESBLs+ kpn	36.5 (46/126)	64.5 (167/259)	<0.001	78.9 (60/76)	49.5 (153/309)	<0.001	55.4 (128/231)	47.6 (50/105)	0.196
CR-kpn	19.0 (24/126)	42.9 (111/259)	<0.001	53.9 (41/76)	30.4 (94/309)	<0.001	38.5 (89/231)	30.5 (32/105)	0.178
PIP-R-pae	14.3 (4/28)	17.6 (9/51)	0.763	47.4 (9/19)	6.7 (4/60)	<0.001	28.9 (13/45)	4.3 (1/23)	0.025
CR-pae	23.5 (8/34)	24.3 (17/70)	0.999	57.1 (12/21)	15.7 (13/83)	<0.001	27.4 (17/62)	10.0 (3/30)	0.065
SAM-R-aba	76.0 (19/25)	73.8 (62/84)	0.999	90.5 (38/42)	64.2 (43/67)	0.003	76.0 (57/75)	67.7 (21/31)	0.468
CR-aba	78.1 (25/32)	75.7 (81/107)	0.999	90.2 (46/51)	68.2 (60/88)	0.004	78.6 (77/98)	38.2 (26/68)	<0.001
MRSA	30.7 (27/88)	52.0 (52/100)	0.005	60.0 (15/25)	39.3 (64/163)	0.080	45.4 (54/119)	34.1 (15/44)	0.216
MRCNS	72.5 (58/80)	89.1 (114/128)	0.004	86.7 (39/45)	81.6 (133/163)	0.510	87.0 (87/100)	82.6 (57/69)	0.510
AMP-R-ENT	45.0 (27/60)	56.9 (62/109)	0.151	57.9 (22/38)	65.7 (67/102)	0.433	50.0 (36/72)	58.8 (40/68)	0.313
VRE	1.7 (1/59)	1.8 (2/109)	0.999	0 (0/37)	2.3 (3/131)	0.999	1.4 (1/73)	1.5 (1/67)	0.999

AMP-R-ENT, ampicillin-resistant Enterococci; CABSI, community-acquired bloodstream infection; CR-aba, carbapenem-resistant A. baumannii; CR-eco, carbapenem-resistant E. coli; CR-kpn, carbapenem-resistant K. pneumoniae; CR-pae, carbapenem-resistant P. aeruginosa; ESBLs+eco, extended-spectrum β-lactamases-producing E. coli; ESBLs+kpn, extended-spectrum β-lactamases-producing K. pneumoniae; HABSI, hospital-acquired bloodstream infection; ICU, intensive care unit; MRCNS, methicillin-resistant coagulase-negative Staphylococci; MRSA, methicillin-resistant S. aureus; PIP-R-pae, piperacillin-resistant P. aeruginosa; SAM-R-aba, ampicillin-sulbactam-resistant A. baumannii; VRE, vancomycin-resistant Enterococci.

Data are percentage (n/N) unless otherwise specified.

### Antimicrobial therapy and outcomes

Most (79.8%, 2,212/2,773) of the patients received appropriate antimicrobial therapies for BSIs. Namely, at least one of the antimicrobial agents used to treat the BSIs was active against the baseline pathogenic isolate in antimicrobial susceptibility testing. Significantly higher percentage of the patients receiving appropriate antimicrobial therapies (63.4%, 1,757/2,773) than the patients receiving inappropriate antimicrobial therapies (42.8%, 240/561) responded well to the treatment. Inappropriate antimicrobial therapies were associated with significantly higher in-hospital mortality rate (18.5%, 104/561) of BSIs than appropriate antimicrobial therapies (7.0%, 155/2,212) (*p* <.01).

## Discussion

It is important to understand the profile and patterns of antimicrobial resistance in BSIs for clinicians to prescribe more proper and appropriate antimicrobial therapies, especially in the context of growing antimicrobial resistance (Musicha et al., 2017). Therefore, this clinical investigation was conducted in the patients confirmed with BSI diagnosis, which is different from the data of isolates from positive blood culture collected from conventional antimicrobial resistance surveillance program. Generally, conventional surveillance cannot exclude all colonizers and contaminants and so cannot identify each of the real pathogens appropriately. In this series, 2,773 cases of BSIs were mostly monomicrobial (97.3%). About two-thirds (67.5%) of the 2,861 strains of pathogens isolated from BSIs were Gram-negative bacteria such as *E. coli* and *Klebsiella*. One-third of the pathogens were Gram-positive bacteria (29.6%) and fungus (2.9%). In contrast, SENTRY Antimicrobial Surveillance Program reported that *S. aureus* and CNS were among the top 3 pathogens of BSIs in various international regions during 1997–2002, and Gram-positive bacteria accounted for 45%–50% of all BSI pathogens. Since 2005, Gram-positive bacteria become less prevalent, and accordingly, the proportion of Gram-negative bacteria goes up slightly among BSI pathogens. Among the top 10 BSI pathogens, the proportion of Gram-negative bacteria increased from 33.5% before 2005 to 43.4% during 2013–2016 associated with higher resistance level (Biedenbach et al., 2004; Musicha et al., 2017; Pfaller et al., 2020). In the present report, the higher proportion of Gram-negative bacteria may be due to the following factors: the primary site of infection was mostly urinary tract, biliary tract, and abdominal infection; CNS isolates were considered as colonizer or contaminants and were thus excluded from analysis; only 37.5% of the BSIs were community acquired so that the Gram-positive pathogens such as *S. pneumoniae* were under-represented (4.5%) in our data.

This investigation revealed that the pathogens of BSIs varied with patient population and clinical setting. *Escherichia coli* and *S. aureus* were more prevalent in CABSIs than in HABSIs, which may be partly due to the fact that there is a higher proportion of urinary tract infections and skin and soft tissue infections in the primary source of CABSIs. *Acinetobacter baumannii* was more prevalent in HABSIs than in CABSIs. Additionally, ICU patients were associated with a higher proportion of *A. baumannii* and CNS isolates identified from BSIs than from non-ICU patients, which suggests such bacteria mostly originated from hospital services, such as invasive procedures. Non-ICU patients were associated with a higher proportion of *E. coli* than ICU patients, which is attributed to the higher percentage of CABSIs in non-ICU patients compared to that in ICU patients. The pathogens of BSIs also varied with age. Specifically, *Enterococci* were more prevalent in old patients, while *S. agalactiae* was more prevalent in newborns.

The BSI pathogens isolated from different patient populations showed different susceptibility profiles. HABSIs were associated with a higher prevalence of carbapenem-resistant *K. pneumoniae*, ESBL-producing *K. pneumoniae*, MRSA, MRCNS, and ampicillin-resistant *Enterococci* than CABSIs. ICU patients were associated with significantly higher resistance rates of *K. pneumoniae*, *P. aeruginosa*, and *A. baumannii*, and a higher prevalence of BSI isolates of carbapenem-resistant *E. coli* and MRSA than non-ICU patients. These findings suggest that the hospital-acquired BSI pathogens, especially in ICU patients, are generally more resistant than CABSI isolates.

The antimicrobial susceptibility of BSI pathogens indicated that about 50% or higher percentage of the *E. coli* and *K. pneumoniae* isolates were resistant to the commonly used broad-spectrum penicillins (including ampicillin-sulbactam), cephalosporins, gentamicin, and fluoroquinolones. This finding suggests that these antibiotics are not appropriate as empirical therapy to treat the BSIs caused by *E. coli* or *K. pneumoniae*, while β-lactam/β-lactamase inhibitor combinations (excluding ampicillin-sulbactam), amikacin, and carbapenems are appropriate treatment options. It is important to note that compared to *E. coli*, a higher percentage of *K. pneumoniae* strains were resistant to piperacillin-tazobactam and carbapenems. The antimicrobial therapy for the BSIs caused by *K. pneumoniae* should be based on susceptibility testing results. Polymyxins and tigecycline inhibited 96%–97% of the *E. coli* and *K. pneumoniae* isolates *in vitro*, but they are not appropriate as empirical therapy to treat BSIs because insufficient clinical evidence is available to support such empirical use (Katz et al., 2016; Zhou C, et al., 2021). Polymyxins and tigecycline should only be prescribed for the targeted treatment of specific pathogens based on susceptibility testing results. In this series of BSI cases, 33.2% and 34.1% of the *K. pneumoniae* isolates were resistant to imipenem and meropenem, respectively, much higher than the CHINET surveillance data in 2016 (15.4% resistant to imipenem and 17.9% resistant to meropenem) (Hu et al., 2017) and in 2020 (21.5% resistant to imipenem and 22.4% resistant to meropenem) (Hu et al., 2021). This may be related to the following conditions. More HABSIs than CABSIs (61.6% *vs*. 38.4%) cases were included in this analysis. HABSI isolates were more resistant than CABSI isolates, especially a higher percentage of carbapenem-resistant *K. pneumoniae* (CRKP) in HABSI isolates. The latest guidelines recommend taking ceftazidime-avibactam, meropenem-vaborbactam, and imipenem-relebactam as first-line treatment and cefiderocol as alternate therapy to treat the BSIs caused by CRKP (Tamma et al., 2021). The high proportion of CRKP in this series of BSI cases also reflects the increasing prevalence of CRE in the last 16 years in China. According to the CHINET report (Hu et al., 2021), the prevalence of CRKP increased from 2.9% to 3.0% in 2005 to the highest 25% to 26% in 2018, and 23% to 24% in 2019–2020. This is generally consistent with the global data (Diekema et al., 2019). The latest guidelines recommend the assay of carbapenemases for precision treatment of CRKP infections (Han et al., 2020; Yu et al., 2020), which is vital for the early diagnosis of CRKP BSIs. More than 80% of the *P. aeruginosa* isolates in this series of BSIs were susceptible to ceftazidime, piperacillin-tazobactam, aminoglycosides, and fluoroquinolones, which can be considered as empirical therapies. The prevalence of imipenem-resistant *A. baumannii* increased from 32.9% in 2005 to 72.9% in 2020 according to the CHINET report. The *A. baumannii* isolates were mostly resistant to carbapenems, β-lactam/β-lactamase inhibitor combinations, aminoglycosides, and fluoroquinolones (generally <40% susceptible), and more resistant to imipenem and meropenem compared with the data of foreign countries (70.6% *vs*. 55.3%) (Tamma et al., 2021). However, the *A. baumannii* isolates were highly susceptible to polymyxins and tigecycline.

The prevalence of MRSA was 42.0% in this series of BSI cases, comparable to the data in other regions (25%–50%) (You et al., 2017). The MRSA isolates were susceptible to vancomycin and linezolid, which can be used to treat MRSA BSIs, alone or in combination with rifampicin if necessary. Oxacillin and clindamycin are still appropriate therapies for the BSIs caused by methicillin-susceptible *S. aureus* (MSSA). The prevalence of vancomycin-resistant *Enterococci* (VRE) was much lower than that in the US (2.7% *vs*. >50%) (Mendes et al., 2016). Nearly half (44.1%) of the enterococcal isolates were resistant to ampicillin, which should not be considered in empirical therapy. Vancomycin and linezolid are still the good choice for treating the BSIs caused by ampicillin-resistant *Enterococci* due to their potent activity.

This multicenter clinical investigation reveals that Gram-negative bacteria especially *E. coli* and *K. pneumoniae* are more prevalent than Gram-positive bacteria such as *Staphylococcus* and fungus such as *Candida* species in the BSI pathogens. The distribution and antimicrobial resistance of BSI pathogens varied with patient population. Specifically, HABSIs and ICU patients were associated with a higher percentage of antibiotic-resistant pathogens than CABSIs and non-ICU patients. The BSI pathogens were highly resistant to the commonly used broad-spectrum penicillins and cephalosporins but relatively susceptible to carbapenems, β-lactam/β-lactamase inhibitor combinations, and vancomycin. The increasing prevalence of CRKP poses a serious challenge for BSI treatment. In this study, among 6,235 blood-culture isolates collected from 5,613 patients from 10 participating cites during CHINET 2016, 1966 episodes were excluded as contaminants, and the majority were isolated with single or dual coagulase-negative *Staphylococci*. One of the most likely reasons for such a high contamination rate is the contamination of the skin surface with colonized bacteria due to irregular skin disinfection during the collection of blood specimens. The findings in this study add more evidence to inform the empirical and targeted precision antimicrobial treatment for BSIs.

## Data availability statement

The original contributions presented in the study are included in the article. Further inquiries can be directed to the corresponding authors.

## Author contributions

YinZ, DZ, and DL designed the study. HF, LL, and YaY, performed the experiments and wrote the manuscript. YuX, YiH, ZC, ZS, YuH, XA, YiX, XZ, YN, JS, YuY, JL, CZ, DS, CW, LH, SB, and YD performed the experiments. All authors contributed to the article and approved the submitted version.

## Funding

This work was supported by the National Key Research and Development Program of China (2021YFC2701800 and 2021YFC2701803), National Natural Science Foundation of China (81861138052), the grants from China Antimicrobial Surveillance Network (Independent Medical Grants from Pfizer, 2020QD049), and Shanghai Antimicrobial Surveillance Network (grant no. 3030231003). The funders had no role in the study design, data collection and analysis, decision to publish, or preparation of the manuscript.

## Conflict of interest

The authors declare that the research was conducted in the absence of any commercial or financial relationships that could be construed as a potential conflict of interest.

The handling editor YWT declared a past co-authorship with the authors FH and YY.

## Publisher’s note

All claims expressed in this article are solely those of the authors and do not necessarily represent those of their affiliated organizations, or those of the publisher, the editors and the reviewers. Any product that may be evaluated in this article, or claim that may be made by its manufacturer, is not guaranteed or endorsed by the publisher.
